# To Be Published in January

**Published:** 1866-12

**Authors:** 


TO BE PUBLISHED IN JANUARY—*The Micro-Chemistry of Poisons*, including their Physiological, Pathological and Legal Relations, adapted to the use of the Medical Jurist, Physician and General Chemist. By Theod. G. Wormley, M. D., Professor of Chemistry and Toxicology in Starling Medical College, and of Natural Sciences in Capital University of Columbus, Ohio. 8vo., 650 pages, with 78 illustrations upon steel and wood cuts; handsomely bound in cloth. Price, $10.

BAILLIÈRE BROTHERS, 520 Broadway, N. Y.

